# Insights into *EGFR* Mutations and Oncogenic *KRAS* Mutations in Non-Small-Cell Lung Cancer

**DOI:** 10.3390/cancers15092519

**Published:** 2023-04-28

**Authors:** Rafael Rosell, Andrés Aguilar-Hernández, María González-Cao

**Affiliations:** 1 Germans Trias i Pujol Research Institute, 08916 Badalona, Spain; 2 IOR, Hospital Quiron-Dexeus, 08028 Barcelona, Spain

Genetic mutations can activate different sets of proto-oncogenes and tumor suppressors genes. However, in lung adenocarcinoma, some combinations of mutations are mutually exclusive, such as mutations in *EGFR* and *KRAS* oncogenes, which are detrimental to cancer cells when combined. The co-expression of mutant *KRAS* and *EGFR* potentiates MAPK signaling through extracellular-signal-regulated kinases (*ERK1/2*), which mediate toxicity, thereby inducing morphological changes and increased micropinocytosis in lung adenocarcinoma cells [1,2]. This Special Issue of *Cancers* features five new articles, four of which focus on *EGFR*-mutant NSCLC patients, while one reports on the characteristics and clinical outcomes of Norwegian *KRAS*-mutant NSCLC patients.

We-Chien Huang and colleagues [3] identified the principal relevance of the MEK/ERK/miR-21 signaling pathway in Osimertinib resistance in *EGFR*-mutant NSCLC cells. The researchers [3] observed ERK reactivation following targeted therapies in NSCLC cells with different genotypes. They also found that triple mutations of *EGFR* sensitized with T790M/C797S amplify ERK signaling and that these cells are sensitive to trametinib, an MEK inhibitor. Christine M. Lovly’s group [4] previously showed that the combination of Osimertinib plus selumetinib (MEK inhibitor) inhibited cell proliferation in a panel of *EGFR*-mutant cell lines. Furthermore, the addition of BKM 120 (a PI3K inhibitor) or dasatinib (an SFK inhibitor) enhanced the effects of Osimertinib. Likewise, a dasatinib–Osimertinib combination was the most effective at inhibiting tumor cell proliferation. Src family kinases (FAK) have proven to be relevant in Osimertinib-resistant *EGFR*-mutant tumor cells [4,5,6], which were also reviewed by Rosell et al. [7]. Another noticeable aspect of Huang et al.’s [3] study is the revelation that Osimertinib-resistant NSCLC cells provoke the transformation of human lung fibroblasts into cancer-associated fibroblasts (CAFs) via the secretion of interleukin-6 (IL-6)-STAT3 signaling. CAFs enhance tumorigenesis by releasing IL-6, IL-8, and HGF and increasing the concentration of miR-21 in NSCLC cells [3]. These findings are in consonance with the seminal description from the group led by Jacqueline F Bromberg [8], wherein the authors identified that *EGFR*-mutant cells produce high IL-6 levels and that the blockade of the IL-6/gp130/JAK pathway led to a decrease in phosphorylated STAT3 levels, but not when dasatinib was used, indicating that the combination of STAT3 and Src/SFK inhibition could be necessary [7]. In addition, following treatment with erlotinib, *EGFR*-mutant NSCLC cells that are becoming resistant undergo an epithelial-to-mesenchymal transition (EMT), wherein IL-6 continues to be expressed independently of the activation of *EGFR* and under the control of TGF-β [9]. It was recently reported that IL6 suppresses T and NK-cell function in EMT-associated EGFR TKI-resistant *EGFR*-mutant cells. In the related study, treatment with IL6 antibodies enhanced antitumor immunity and the efficacy of immune checkpoint anti-PD1 inhibitor [10]. Controversy surrounds the function of miR-21, which was shown to be oncogenic in the aforementioned study by Huang et al. [3]. However, a previous study by Amyn A Habib’s group offered a different view, suggesting that *EGFR* mutations suppress TNF mRNA levels by inducing the expression of miR-21, which leads to a decrease in TNF mRNA levels. Conversely, *EGFR* inhibition (e.g., with erlotinib) leads to a loss of miR-21 and an increase in TNF mRNA levels and, consequently, TNF-induced NF-kB activation [11].

Trever G Bivona and colleagues reported that NF-kB signaling causes resistance to *EGFR* inhibitors. They found that the loss of NFKB1A, which suppresses NF-kB signaling, is associated with poor progression-free survival. Furthermore, suppressing the NF-kB pathway enhances the response to *EGFR* TKIs in *EGFR*-mutant models [12,13]. Gong et al. [11] suggested that the co-inhibition of *EGFR* and TNF (etanercept) could increase the therapeutic benefit in *EGFR*-mutant NSCLCs. Therefore, the aforementioned study by Huang et al. [3] reinforces previous knowledge in the field of *EGFR*-mutant tumors and highlights the need to further understand ERK signaling and the role of miR-21 in light of the apparently divergent findings of Huang et al. [3] and Gong et al. [11]. 

According to the study conducted by Huang et al. [3], IL-6 plays a significant role in both the downstream effects and upstream origins of ERK signaling in naïve and resistant *EGFR*-mutant NSCLCs. The identification of these factors raises tantalizing questions. However, prior to the discovery of *EGFR* mutations, Rafaella Sordella and colleagues had already identified that adequate treatment for mutant-*EGFR* requires pharmacological inhibitors of the signal transducer and activator of transcription (STAT3) and Akt signaling pathways [14].

Also included in this Special Issue of *Cancers*, Yan-Jei Tang and colleagues [15] propose the important clinical utility of using Osimertinib after the presence of the acquired *EGFR* T790M mutation instead of administering Osimertinib as a first-line treatment for *EGFR*-mutant NSCLCs. This is a complex dilemma that involves subtle intricacies in clinical management and underlying biological mechanisms. According to the ASCO Living Guidelines, *EGFR*-mutant patients (L858R/exon 19 deletions, with or without concomitant T790M) with PS0-2 should be treated with Osimertinib [16]. However, a Dutch study suggests that stage IV *EGFR*-mutant NSCLCs with exon 19 deletion presented better survival than those with the L858R mutation [17]. Additionally, *EGFR*-mutant patients with an exon 19 deletion and brain metastases present better survival with Osimertinib compared to other EGFR TKIs [17]. The retrospective analysis conducted by Tang et al. [15] also found that brain metastasis together with T790M were associated with Osimertinib benefit. 

The FLAURA trial clearly revealed that in patients with previously untreated NSCLC with *EGFR* mutations, Osimertinib resulted in longer overall survival compared to gefitinib or erlotinib. However, a subgroup analysis found that this advantage was not seen in Asian patients or in patients with the L858R mutation (hazard ratio, 1.00; 95% CI 0.71–1.40) [18]. 

While the lack of an overall survival advantage in Asian patients deserves further investigation to uncover its underlying genetic reasons, the advantage of Osimertinib with respect to *EGFR* exon 19 deletions has been associated with an enrichment in the occurrence of T790M mutations [19].

In contrast, the probability of developing T790M mutations in *EGFR* exon L858R mutant NSCLCs is lower. Additionally, this type of mutation is often associated with TP53 mutations and 3q23 amplification, of which the latter contains the MRAS gene that is part of the SHOC2 phosphatase complex involved in resistance to EGFR TKIs [20]. Likewise, Tang et al.’s study [15] reinforces the previous clinical and biological evidence suggesting that sequential therapy with EGFR TKIs, starting with first- or second-generation drugs and then switching to a third-generation drug such as Osimertinib, could be an effective treatment strategy and should be taken into consideration.

The third study in this Special Issue was conducted by Jung Hee Cho and colleagues [21]. The investigators identified LPIN1, an Mg^2+^-dependent phosphatidic acid phosphatase (PAP) enzyme that converts phosphatidic acid to diacylglycerol, a precursor of triacylglycerol and phospholipids. In this study, the authors observed that gefitinib treatment induced LPIN1 expression, thereby enhancing diacylglycerol concentrations in EGFR-TKI-resistant H1650 cells followed by activating protein kinase C-**δ** and NF-kB. This finding sheds new light on the role of NF-kB activity in resistance to EGFR TKIs. The authors also found that the loss of LPN1 expression sensitizes *EGFR*-mutant NSCLC cells to gefitinib in vivo. The investigators used propranolol as an LPIN1 inhibitor [21] and attained intriguing results, which were similar to those of previous studies conducted by John Heymach’s group. These studies have shown that β2-adrenergic receptors can upregulate IL6, leading to resistance to EGFR TKIs in *EGFR*-mutant NSCLC cells. Propranolol was used as a β-blocker, which efficiently inhibited resistance to EGFR TKIs [22]. The relevance of diacylglycerol kinase α-phosphatidic acid-phosphodiesterase activity in activating the mTOR pathway has been demonstrated in glioblastoma and other tumors [23]. Based on these findings, it has been postulated that phosphodiesterase inhibitors could be applicable in *EGFR*-mutant NSCLCs [24].

In this Special Issue of *Cancers*, Cansouline et al. report a revisitation of the position of EGFR TKIs in early surgically resected *EGFR*-mutant NSCLCs [25]. The study reinforces the current perception that, overall, EGFR TKIs increase event-free survival. However, the review cautions that the benefit of overall survival deserves further analysis. The phase III ADAURA study provides updated results on *EGFR*-mutant stage IB-IIA NSCLC after complete resection, showing a 4-year disease-free survival rate of 73% with Osimertinib and 38% with a placebo (hazard ratio 0.27). Furthermore, it supports the use of adjuvant Osimertinib in resected *EGFR*-mutant NSCLCs due to improved central nervous system disease survival [26]. 

However, as seen in stage IV NSCLC patients and *EGFR*-mutant cells, the mechanisms of resistance mentioned earlier could also occur in the adjuvant and neoadjuvant settings. Additionally, the promigratory signal Src-homology-2 domain-containing phosphatase-2 (SHP2, encoded by the PTPN11 gene) activates Src family kinases (SFK). We previously reported that high levels of SHP2 mRNA correlated with poor progression-free survival and overall survival in metastatic *EGFR*-mutant NSCLC cells treated with EGFR TKIs [27]. We also observed that elevated SHP2 mRNA levels were associated with recurrence in resected *EGFR*-mutation-positive lung adenocarcinomas but not in the *EGFR* wild-type [28]. Ito et al. [28] reported that EGFR TKI inhibitors increase SHP2 activation and limit the efficacy of EGFR TKIs. Our observations suggest that SHP2 may enhance the efficacy of adjuvant EGFR TKI treatment [28].

The fifth article included in this Special Issue by Sissel Gyrid Freim Wahl [29] shares similarities with an array of studies that document the frequencies of *KRAS*-mutant NSCLCs, including the different genotypes of druggable *KRAS* G12C and non-G12C mutations, while scrutinizing potential variables that could shed light on the effects of anticancer therapies. Wahl and colleagues [29] add further valuable clinical data to this field. Other recent studies in this regard have reported similar findings in Asian and Hispanic patients, reinforcing the fact that the frequency of *KRAS* mutations is lower in these populations than in Caucasian NSCLC patients [30,31]. The rapid development of *KRAS* G12C inhibitors presents new opportunities and challenges for researchers investigating the mechanisms of resistance to sotorasib, adagrasib, and SHP2 inhibitors, which appear to be analogous to those identified in other targeted therapies such as EGFR TKIs in *EGFR*-mutant NSCLCs [32].
